# Quantification Method for Electrolytic Sensors in Long-Term Monitoring of Ambient Air Quality

**DOI:** 10.3390/s151027283

**Published:** 2015-10-27

**Authors:** Nicholas Masson, Ricardo Piedrahita, Michael Hannigan

**Affiliations:** Department of Mechanical Engineering, University of Colorado at Boulder, 427 UCB 111 Engineering Drive, Boulder, CO 80309, USA; E-Mails: ricardo.piedrahita@colorado.edu (R.P.); hannigan@colorado.edu (M.H.)

**Keywords:** electrolytic, amperometric, air quality, pollution, monitor, quantification model

## Abstract

Traditional air quality monitoring relies on point measurements from a small number of high-end devices. The recent growth in low-cost air sensing technology stands to revolutionize the way in which air quality data are collected and utilized. While several technologies have emerged in the field of low-cost monitoring, all suffer from similar challenges in data quality. One technology that shows particular promise is that of electrolytic (also known as amperometric) sensors. These sensors produce an electric current in response to target pollutants. This work addresses the development of practical models for understanding and quantifying the signal response of electrolytic sensors. Such models compensate for confounding effects on the sensor response, such as ambient temperature and humidity, and address other issues that affect the usability of low-cost sensors, such as sensor drift and inter-sensor variability.

## 1. Introduction

Electrolytic or amperometric sensors are one of several types of sensors commonly cited when discussing low-cost air monitoring technologies. They are ideally suited for many air quality applications and strike a good balance when considering price, size, durability, selectivity, sensitivity and response time. Using electrolytic sensors in ambient monitoring, however, is still a challenge. Electrolytic sensors have been deployed in sensor network projects [1,2,3], but such projects produce little usable data without proper quantification of the raw signal into predicted pollution concentrations. Such quantification schemes should not only address the translation of raw signal output to units of pollution concentration, but also provide an appropriate scope of use and insight for effective and efficient calibration procedures.

Previous work has addressed the underpinnings of electrochemical cell behavior [4,5], but few [6] offer practical models for implementing electrochemical sensors in ambient monitoring. This work offers an approach to using electrochemical sensors, building from first principles of electrochemical behavior, while remaining mindful of the broad variable space within which these devices operate. The data used in deriving and testing the models in this work were collected over a nine-month period of collocation with reference instruments. Variables were independently explored by binning the data into subsets, effectively holding constant those factors in the multivariate response that are not under investigation. This is a feasible approach given the volume of data and the number of repeated observations at variable combinations of interest.

Several companies offer electrolytic cells for measuring ambient criteria pollutants. This study uses the Alphasense line of B4 sensors. This sensor line provides an extra electrode (an auxiliary electrode) that can be used in compensating for baseline changes in the sensor signal. This work also offers a method for correcting an electrolytic sensor signal using temperature and humidity observations, which is useful for other, less expensive sensors, which do not have an auxiliary electrode. Moreover, the approach adopted in this study is applicable when considering most types of low-cost air sensors, given the common attributes, such as intersensor variability, drift, signal noise and response to confounders, specifically temperature and humidity.

## 2. Study Design

Four UPODembedded systems [7] were collocated with regulatory instruments at the Colorado Department of Health and Environment monitoring site (CAMP) at 2105 Broadway St. Criteria pollutants CO, NO/NO_2_ and O_3_ are measured at the CAMP station using a CO analyzer Thermo Electron 48c, a nitrogen oxides analyzer Teledyne 200E and ozone analyzer Teledyne 400E, respectively. Each UPOD system included four electrolytic sensors. Three of the UPODs had Alphasense B4 sensors for NO_2_, CO, NO and O_3_. The work presented here addresses the sensor signals from the Alphasense B4 NO sensors. Temperature and humidity were measured using a Maxdetect RHT03 (also known as DHT22) sensor. Barometric pressure and a second temperature reading were taken using a Bosch BMP085 sensor.

The UPODs were placed within approximately three meters and at the same level as the sample inlet to the reference instruments. The sensors were sampled once every 15 s and averaged into minute readings. Data were collected from December of 2013 to November of 2014.

### 2.1. Instrumentation

The sensor signals were measured and stored using the open-source UPOD platform. The UPOD is an embedded system for data acquisition and data logging/transmission. It accommodates a number of low-cost environmental sensors, among them the electrolytic sensors used in this study.

Unlike many other sensors, the quality of data from electrolytic sensors depends on the circuitry as much as it does on the sensor itself. The sensitivity of the Alphasense NO-B4 sensor, for example, is 0.5 to 0.85 nA/ppb. The target span of 0to 100 ppb NO, a common ambient range, would translate at most to a raw signal span of 0 to 85 nA. Amplifying and resolving such miniscule currents requires robust instrumentation with a high degree of noise attenuation.

The potentiostat designed for the UPOD is roughly based on an Analog Devices publication for gas detection circuitry. All operational amplifiers in the circuit are Analog Devices ADA4528 ultra-low noise, zero-drift op amps. The transimpedance amplifier measuring the working electrode current has a 100 kohm , 0.1% tolerance feedback (gain) resistors, effectively converting 100 µV per nA current. A Microchip MCP3424 18-bit sigma-delta analog to digital (ADC) converter with least significant bit/resolution voltage of 15.625 µV measures the voltage drop across the gain resistor. The digital signal logged by the UPOD is corrected in post-processing for temperature and offset error in the MCP3424 conversion. The typical input voltage offset error of 0.3 µV and input offset current of 440 pA for the transimpedance amplifier op amp is assumed negligible. The 3.2-pF sampling capacitor and 2.25-Mohm input impedance of the MCP3424 is assumed to have little to no bias effect on the ADC reading due to the circuits’ low-pass output filter. The working electrode of the electrolytic sensor is sampled at a sufficiently low rate as to be treated as a DC signal. The printed circuit board (PCB) layout was designed so as to minimize current loops and includes proper decoupling of power supplies in the potentiostat design.

Four potentiostat circuits were mounted on the same PCB for this study. Each potentiostat accommodates one Alphasense B4-type sensor and has two channels, one for the main electrode and another for an auxiliary electrode. The auxiliary electrode is unique to the B4 line of Alphasense sensors and is intended for the correction of the main electrode current to all interference factors.

The baseline performance of the potentiostat circuit was evaluated by placing the potentiostat PCB in a sealed enclosure with four Alphasense B4 CO sensors. Mass flow controllers were used to flow regulatory grade Air Liquide zero grade air through the enclosure. The sensors were left to stabilize for approximately seven hours. The standard deviation of the main electrode signal, sampled once every fifteen seconds, was calculated for a thirty-minute period following the stabilization period. The standard deviation for all four sensors was less than the 15.625 µV LSB resolution of the ADC. The standard deviation was also well below the detection limit of the sensors, as noted in the specification sheets. This test implies low intrinsic noise in the sensor/potentiostat system. It does not account for other sources of noise, such as inductive coupling with electromagnetic (EMF) sources, variability due to Seebeck junction voltages, and parasitic noise due to moisture condensation.

### 2.2. Sensor Fundamentals

Electrolytic sensors function by reacting a target analyte and producing a proportional current. Their most basic form consists of two electrodes (an anode and a cathode) submerged in an electrolyte. A current signal is generated when a redox reaction takes place. The anode is oxidized, and the cathode is equally reduced. Charge moves between the electrodes via ionic conduction and produces a current proportional to the overall reaction rate. The electrode type, electrolyte type and working electrode voltage potential govern which chemical reactions take place in the electrolytic cell. Many factors affect the overall reaction rate in an electrolytic cell. The overall reaction rate is kinetically controlled if the chemical reaction rate is the limiting process. If the chemical kinetics are fast, then physical processes, mainly diffusion, are the rate-limiting processes.

### 2.3. Data Interpretation

In this study, it will be assumed that nothing is known *a priori* about the sensor other than the specifications listed in the manufacturer datasheet. This approach is adopted to parallel the process in which a third party might characterize a sensor and implement a quantification model with limited knowledge from the manufacturer. Experimental data will be used in determining those factors that must be included in a model describing the relationship between the sensor signal (current) and the concentration of the target analyte (environmental pollutant).

The performance standards of low-cost sensor data should be determined given the context in which it will be used and the nature of the questions one hopes to answer. It is often most important to have well-characterized uncertainty in your sensor readings and to understand the significant confounders that corrupt your data. A high-cost instrument will often cite measurement precision or uncertainty as a +/− value irrespective of the environment in which the reading is taken. With low-cost sensors, the reading uncertainty is very much a function of external factors, a function that must be characterized in any robust monitoring implementation.

One approach to data quality assurance adopted in this study is to eliminate data that do not meet predetermined criteria. For example, there may exist a variable space in which the sensor signal cannot be quantified, say times of high temperature or humidity, or in which observational data show such high variability (signal-to-noise) that the data can no longer assess the target question. Those implementing quantification models for electrolytic sensors should assess for themselves if there exists certain operating conditions in which the predicted concentrations no longer suit the accuracy and certainty necessary for a given application. In this study, it was determined that the Alphasense B4 NO sensor often produced unpredictable responses during periods of humidity above approximately 75%, as evidenced by Figure 8, Figure 9, Figure 10 and Figure 11. Filtering the data to eliminate readings that exceed certain criteria can help to maintain overall data quality. Doing so decreases the overall number of samples and possible monitoring variable space, but this is a practical limitation of working with low-cost sensors.

Theory and observation inform the derivation of a model for quantifying the sensor signal when data fall within an acceptable variable space. Observational data and empirical modeling further quantify the joint error distribution and measurement uncertainty as a function of confounding environmental factors.

## 3. Model

Over the span of typical ambient air pollution concentrations, the Alphasense sensor signal current exhibits great linearity with respect to concentration, as cited in the Alphasense specification sheet and verified in controlled laboratory experiments. This suggests that the target analyte’s overall reaction rate is limited by diffusion rather than chemical kinetics. This is supported by the Alphasense Application Note AAN 104 [8] and is common among all makes of electrolytic cell that are designed for linear response characteristics. This is highly desirable, as most of the complexities of the chemistry can be neglected in the model derivation. The effect of cross-sensitive species on the sensor response can be significant, as quantified in the specification sheet. These effects can be corrected for when other reference measurements are taken in conjunction with the electrolytic sensor or in principle when using an array of electrolytic sensors with sufficiently different cross-sensitivities. The work presented here does not take any of these measurements into account and treats the electrolytic sensor as an isolated device with temperature and humidity as the only collocated measurements. It is assumed that ephemeral instances of cross-sensitive species will not significantly affect the model fitting, as data were collected over a long period of time. More persistent sources of cross-sensitive species, like ozone, can have the effect of increasing the prediction uncertainty and should be considered a source of error when assessing the model fit statistics. Another important consideration is the covariance of certain diurnal cross-sensitive species with temperature, which can influence not only the random model error, but also confound the nature of the temperature correction itself. In this work, cross-sensitivity is not incorporated in the model derivation for lack of applicability if devices are not collocated with other sources of measurement, but it is a natural continuation of the derivation should other sources of measurement for cross-sensitive species be present.

In the most general terms, several diffusion processes govern the transport of analyte from the ambient environment to the electrode.

**Figure 1:**
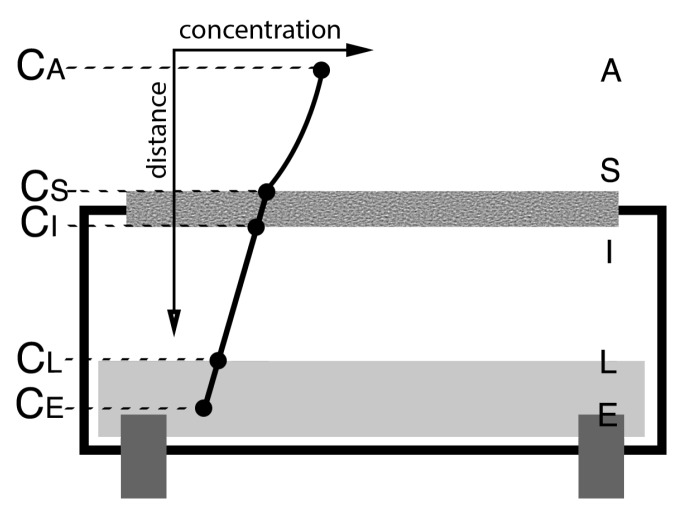
Boundary points in an electrolytic cell denoting important physical interfaces.

Figure 1 depicts a simple electrolytic cell. The net transport of analyte in the ambient environment can be reduced into four simple diffusion processes. First is the transport from the ambient environment (A) to the cell membrane surface (S). Second is the transport across the surface from (S) to (I). Third is the transport from the inner membrane surface (I) to the electrolyte liquid surface (L). Fourth is the transport from the liquid surface (L) to the electrode surface (E), where the final reaction takes place. It is assumed that the net transport is sufficiently slow, such that the transport across the gas-liquid interface is in equilibrium. The concentration of dissolved analyte in the liquid is considered to be proportional to the partial pressure of the analyte in the gas phase, as per Henry’s law (the coefficient of proportionality being Henry’s law constant). Fick’s law describes the diffusion in each of the four layers:

(1) $$J=-D\frac{\partial C}{\partial x}$$

where *D* is the diffusion coefficient, *C* is the analyte concentration at a given point in space, *J* is the flux and ∂C/∂x is the gradient of analyte concentration in a direction *x*. A very simple model can be derived by solving for the flux as a function of the ambient concentration given the four, coupled equations:

(2) $$J=D_{1}\left(\frac{C_{A}-C_{B}}{x_{1}}\right)$$

(3) $$J=D_{2}\left(\frac{C_{B}-C_{C}}{x_{2}}\right)$$

(4) $$J=D_{3}\left(\frac{C_{C}-C_{D}}{x_{3}}\right)$$

(5) $$J=D_{4}\left(\frac{C_{D}-C_{E}}{x_{4}}\right)$$

These equations assume the sensor is operating at steady state (*J* is constant across the four layers) and assume that the gradient is predominantly along one direction, between two concentrations separated by a characteristic distance $x_{n}$. Assuming a close to zero analyte concentration at the electrode surface ($C_{E}∼0$) and solving for the flux as a function of the ambient concentration give:

(6) $$J=G*C_{A}$$

where *G* is a very complex coupling of the diffusion coefficients and the characteristic distances. Defining a normalized diffusion constant $\Psi_{n}\equiv D_{n}/x_{n}$ gives *G* as:

(7) $$G=\frac{\sum_{n=2}^{4}\Psi_{n}}{\Psi_{2}\Psi_{3}+\Psi_{2}\Psi_{4}+\Psi_{3}\Psi_{4}}*\left[1-\frac{\Psi_{2}}{\Psi_{1}\left(\Psi_{2}+1\right)+\Psi_{2}}\right]$$

There is little sense in using the complex value *G* in a sensor quantification model, especially given the simplicity of the diffusion model from which it was generated. *G* does provide some insight, however, into the sensor behavior. The normalized diffusion coefficient $\Psi_{1}$, which is inversely proportional to the characteristic distance $x_{1}$, only appears in the denominator of the far right term, implying an increase in overall flux when $x_{1}$ decreases. Unlike the other characteristic distances, $x_{1}$ is not necessarily constant or close to constant, as it will change with the air’s fluid boundary layer thickness over the sensor. The transport of analyte in a fluid (air) is affected by the advection of gas (bulk transport and turbulence) in addition to diffusion. This advection can be assumed to be minimal within the sensor (stagnant air in the electrolyte head-space). External to the sensor, between the membrane surface (S) and the ambient far-field (A), both advection and diffusion will govern transport. Only within the viscous boundary layer of the airflow over the sensor is diffusion dominant and is the system of Equations (2)–(5) valid. The concentration of gas at the viscous boundary layer is generally of the same concentration (if not defined to be) as the environment one wishes to sample. As such, the characteristic distance $x_{1}$ can be defined as the height of the viscous boundary layer over the sensor, which in turn is affected by external air flow. The higher the velocity of air flow over the sensor, the smaller the distance $x_{1}$ and the greater the flux (increase in sensitivity). Herein lies the justification for using small PCB fans to drive flow directly onto the sensor orthogonal to the membrane surface.

**Figure 2:**
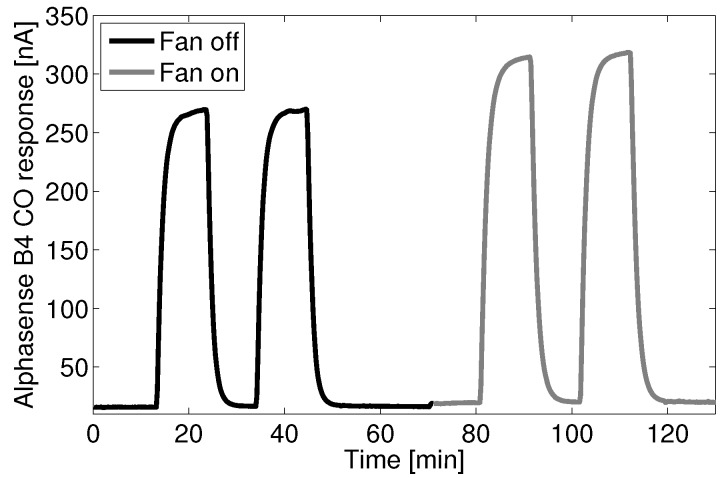
Response of the Alphasense B4-CO sensor to delta steps in CO gas, with and without a fan.

Figure 2 shows the increase in sensitivity of one Alphasense B4 carbon monoxide (CO) sensor when exposed to four instances of 4.8 ppm CO. Four Alphasense B4-CO sensors were tested simultaneously with equal responses. The two first instances were with the fans turned off, and the second two instances were with the fan turned on. The CO concentration was controlled using mass flow controllers with a zero air and 1000 ppm CO gas standard. A bubbler was used to raise the humidity to approximately 30% relative humidity (Rh) . The sensor and potentiostat were placed in a five-liter enclosure with an inlet connected to the mass flow control system. Full recovery of the sensor to its zero current state is observed between consecutive elevated carbon monoxide events.

The presence of the fan increased the sensitivity of the sensor by approximately 17%. A shift in the baseline value of 17% can also be observed when the fan was turned on. This likely indicates an increase in flux of not only the analyte, but also those species present in the ambient environment that contribute to the sensor’s “zero current”.

### 3.1. Sensitivity Dependence on Temperature

**Figure 3:**
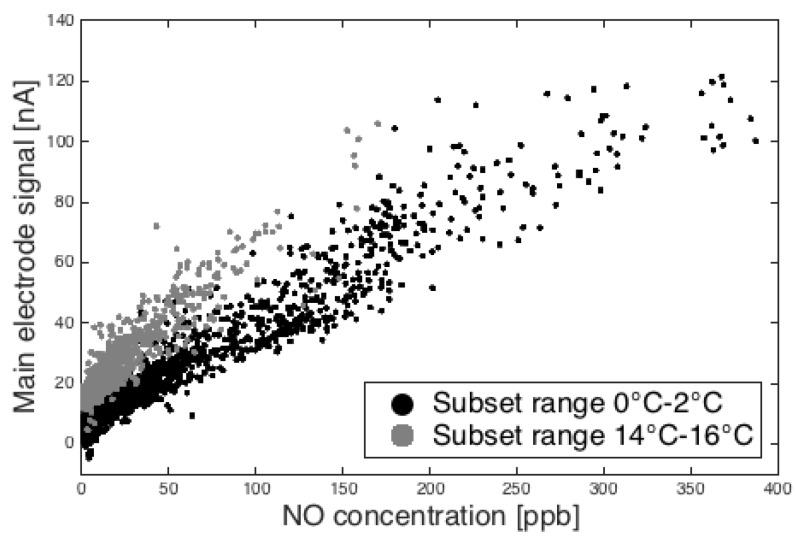
Main electrode response *versus* reference NO measurements illustrating the temperature effect for two subsets of observations in ambient temperature.

As previously noted, the sensitivity of the sensor is proportional to the diffusion-limited flux of analyte to the electrode surface. Figure 3 shows ambient data subsets from January binned into observations that fall between 0 to 4 °C and 12 to 16 °C. A noticeable baseline offset (later discussed in the zero current section) and slope (sensitivity) are apparent between the two groups.

**Figure 4:**
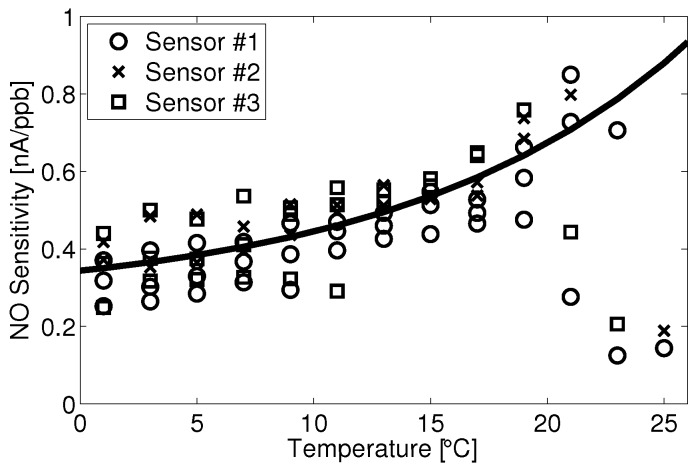
Alphasense B4-NO sensitivity at different ambient temperatures from December to February.

Unfortunately, the *G* term is very complex and does not provide practical means for relating temperature and sensitivity. The gas diffusion terms have a temperature dependence in proportion to $T^{\frac{3}{2}}$ (Chapman–Eskog); the liquid diffusion terms are a function of the change in viscosity with temperature, which can be described by various exponential models, and Henry’s coefficient can also be described to change exponentially in temperature (Van ’t Hoff). Due to this complexity, a fit to empirical data was used to assess the link between temperature and sensor response. An equation of the form Equation (8) was found to fit the data well, as evidenced by Figure 4.

(8) $$S=a_{1}+a_{2}*e^{\left(a_{3}*T\right)}$$

Figure 4 shows the slopes of linear fits of current *versus* concentration (as in Figure 3) against their respective temperature bins. Data are shown for all three NO sensors for the months of December, January and February. The outliers are due to periods of high humidity (e.g., precipitation events), which were not filtered out of the data before fitting current *versus* concentration for each temperature bin. These fits provide the model component for the temperature effect on the sensitivity of the sensor.

The equation coefficients for the fit shown in Figure 3 can be found in Table 1. The equation fit was applied over the aggregate set of data from the three sensors and serves as an initial guess for the optimization discussed in Section 4.

**Table 1:** Equation coefficients for Alphasense B4-NO sensitivity as a function of temperature.

$a_{1}$	$a_{2}$	$a_{3}$
0.263	0.081	0.081

### 3.2. Baseline: “Zero Current”

Ideally, the electrolytic cell would only produce a current in the presence of the target analyte. In reality, the cell has a small reactivity to other ambient gas species, which produces a zero current even in the absence of the target analyte. The chemical reactions that drive the zero current are unknown. As such, it is necessary to empirically model the zero current.

**Figure 5:**
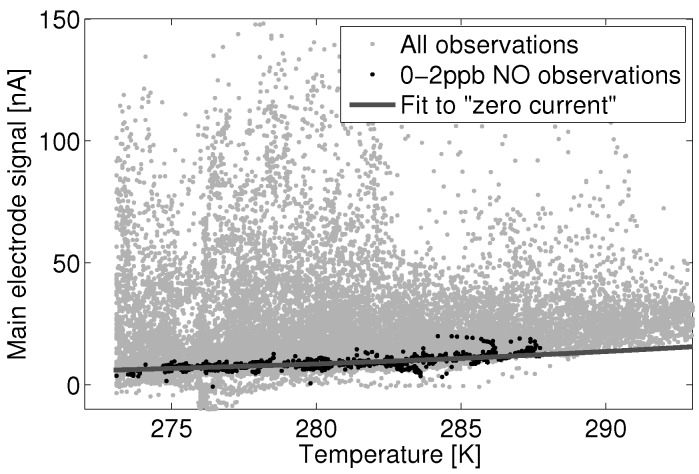
Alphasense B4-NO zero current subset within all NO reference observation and its dependence on temperature.

Figure 5 shows January data for the nitric oxide (NO) main electrode from one UPOD. All observations matching a reference instrument reading of zero to two parts-per-billion (ppb) were separated from the data and considered to be observations of near-zero NO concentration.

These data show a noticeable increase in the main electrode signal with temperature. This is synonymous with an increase in the overall reaction rate of those reactions contributing to the “zero current” signal. A function of the form:

(9) $$a_{1}*e^{\frac{a_{2}}{T}}$$

was fit to the 0 to 2 ppb data subset, where *T* is the measured ambient temperature in Kelvin. This function is of the same form as that of the Arrhenius rate expression, where *k* is the reaction rate constant for a given reaction, $E_{a}$ is the reaction activation energy, *R* is the Boltzmann constant and *T* is the temperature in Kelvin:

(10) $$k=e^{\frac{-E_{a}}{RT}}$$

The Arrhenius rate expression defines the reaction rate constant *k* for a generic balanced reaction of the form:

(11) c*C+b*B→P

Here, the reactant of concentration *C* of stoichiometric coefficient *c* reacts with the reactant of concentration *B* of stoichiometric coefficient *b* to form the product of concentration *P*.

With a reaction rate *r* defined as the change in product over time $\frac{dP}{dt}$ and calculated given:

(12) $$r=k*{\left[C\right]}^{c}*{\left[B\right]}^{b}$$

Although the nature of the reactions and reactants is unknown, it can be assumed that the overall reaction rate takes the general form r=a*k where a=[C][B]. The net zero current is proportional to the sum total of all independent contributing reactions:

(13) $$I_{o}=\sum_{n=1}^{N}a_{n}*k_{n}$$

In principle, a large number of independent reactions, *N*, would give rise to a zero current function of arbitrary shape. By using a single exponential fit to the data with a constant value of *a*, it is assumed that the reactants giving rise to the zero current are near constant concentration in the ambient environment, and there is a limited number of total reactions. This approximation is also appropriate under the assumption that the variability in the concentration and number of total reactions (contributing to the zero current) is stochastic in nature and can be approximated by a global model of a single constant-coefficient exponential term. The proportionality of the zero current to the overall reaction rate is reflected in this constant, *a*, given:

(14) $$I_{o}\propto a*k\to I_{o}=\tilde{a}*k$$

**Figure 6:**
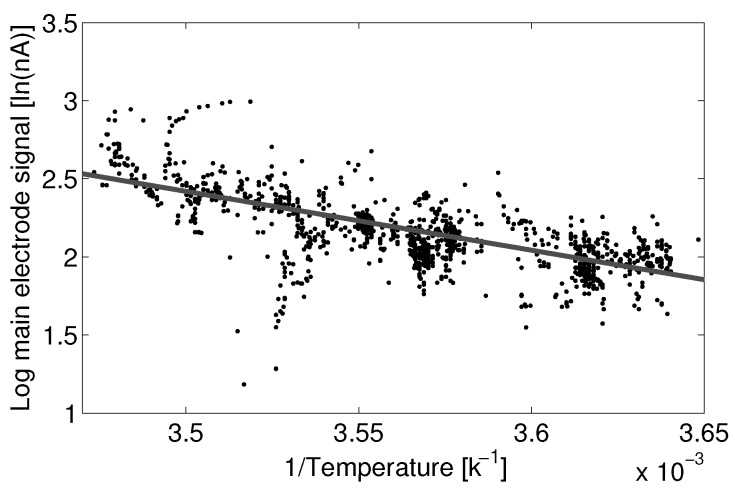
Linear fit to variable transforms of current and temperature for the Alphasense B4-NO main electrode zero current against temperature.

Fitting Equation (9) to the 0 to 2 ppb subset of Sensor 1 gives a fit with RMSE of 1.78 nA. Figure 6 shows this fit as a linear regression of the inverse of temperature against the natural log of the main electrode current. The model coefficients $a_{1}$ and $a_{2}$ for the fit to Sensor 1 are $6.05\times 10^{6}$ and $-3.77\times 10^{3}$, where temperature *T* in Equation (9) is in units of Kelvin.

**Figure 7:**
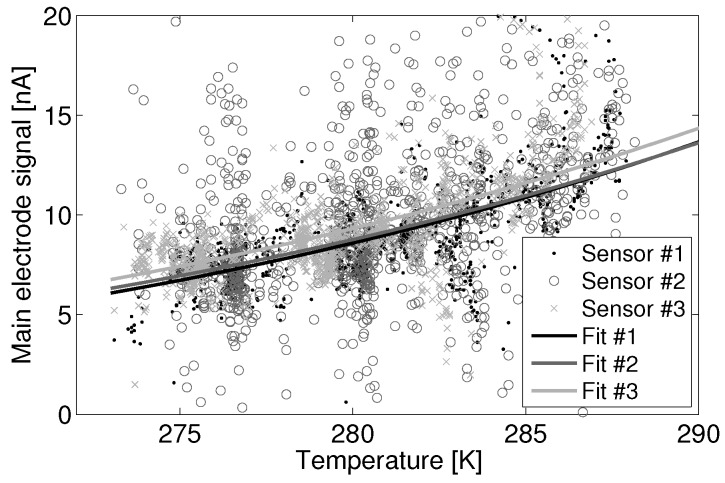
Fit of the main electrode zero current subset against temperature for three Alphasense B4-NO sensors.

Figure 7 shows good inter-sensor agreement for the three collocated NO sensors during the month of January. If inter-sensor variability of several nA is acceptable, then these data suggest that a generalized model can be used among sensors of a given type without an inter-sensor correction factor. This has highly desirable implications for the ease of deployment and calibration. Please note that significant noise is present in Sensor 2, likely due to a faulty power supply, as evidenced by Figure 7.

It can be seen that the fits for Sensor 1 and Sensor 3 are within a nA of each other over the illustrated temperature range. Referencing Figure 4, the sensitivity of the sensors at mid-range temperatures (15 °C) is approximately 0.5 nA/ppb. Thus, the inter-sensor variability between Sensor 1 and Sensor 3 is less than a part-per-billion. The fit for noisy Sensor 2 is closer to a 2-nA or 1-ppb difference from Sensor 1 and Sensor 3.

### 3.3. Humidity

**Figure 8:**
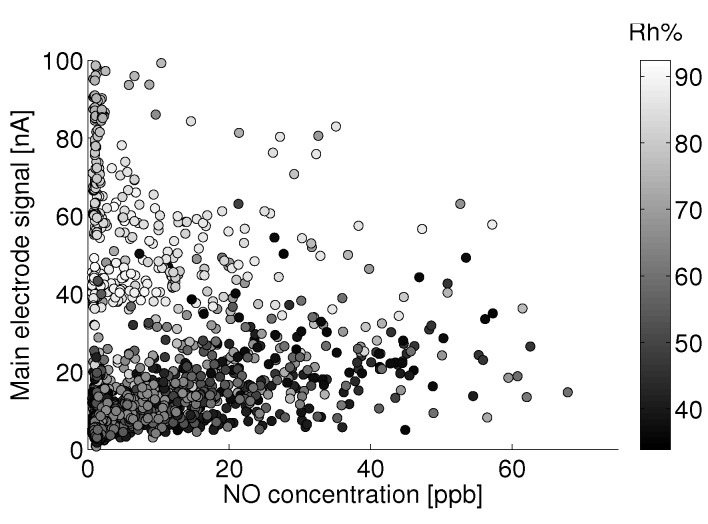
Humidity grayscale-coded scatter plot of the main electrode signal against reference NO measurements between 18 °C and 20 °C for the month of August.

**Figure 9:**
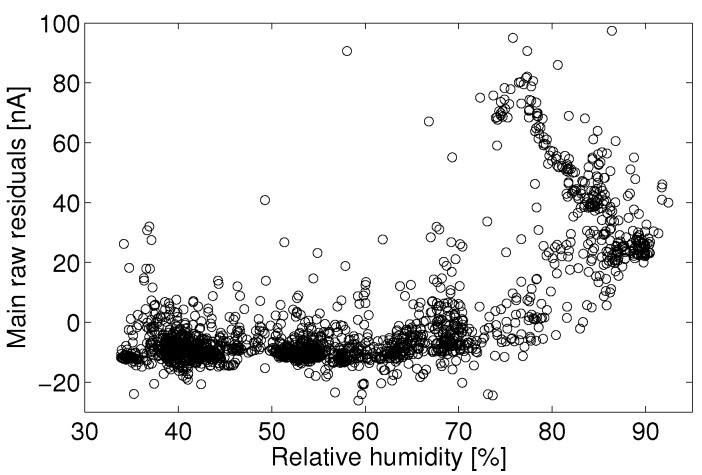
Least-square linear fit residual for observations from 18 °C to 20 °C.

**Figure 10:**
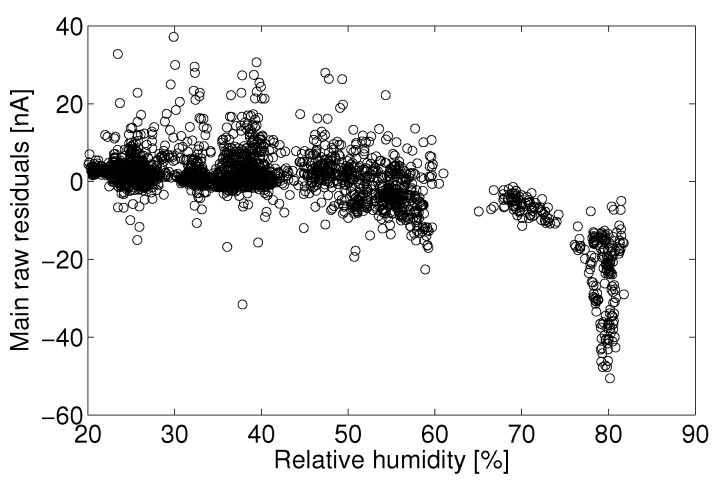
Residual error for a linear least-square fit of the main electrode current *versus* reference NO measurements between 18 °C and 20 °C for the month of August.

Humidity has an unpredictable affect on the electrolytic sensor response. Figure 8 shows the main electrode signal against NO concentration for all observations between 18 °C and 20 °C for the month of August. This temperature range was chosen because of the large number of observations within this temperature bin for the given month. Figure 9 shows the raw residual *versus* humidity for a linear fit to those data. Here, raw residual refers to the error between these data and the linear fit. It can be seen that a significant positive current arises for relative humidity levels above approximately 75%. The confounding of the main electrode signal by high humidity levels is not consistent and repeatable, as evidenced by Figure 10, which shows the same linear fit residual between 2 °C and 4 °C for the month of January.

These data would suggest that the effect of humidity should not be treated analogously to that of another analyte as with the zero current contributors. If this were the case, there would exist a consistent main electrode response as a function of he water mole fraction (absolute humidity). In the present case, the error due to humidity becomes significant around 75% Rh and shows little effect at lower humidity.

With the lack of a robust humidity correction and for the sake of model fitting, all observations above 75% Rh will be discarded to avoid adverse biasing effects. Parallel analysis will take place with the entire dataset using the auxiliary electrode as a correction mechanism. Lab experiments have shown that the main electrode signal increases significantly when a rapid change in humidity (10 s of %) occurs. The sensor is slow to reach a separate steady state after the change in humidity (>10 min). A more complex analysis might consider the effect of the baseline signal on a frequency decomposition of the humidity signal. For example, a consistent correction may exist for slow diurnal changes in humidity, whereas quick changes in humidity (e.g., a precipitation event) may require a different correction. It is also possible that the humidity effect seen here is in part due to condensation on the potentiostat electronics. Such an analysis will not be addressed here.

### 3.4. Auxiliary Electrode

Enhanced correction is possible when an electrolytic cell is equipped with an auxiliary electrode, as with the Alphasense B4 line. The auxiliary electrode on the B4 sensor is meant to respond to all of the same confounders as the main electrode, yet not respond to the target analyte. In other words, the auxiliary electrode should respond to all non-target signals that affect the main electrode and for which one hopes to correct the main signal. It is necessary to first investigate if the main electrode and the auxiliary electrode respond similarly to shared confounders before using the auxiliary electrode to correct for the main electrode signal.

**Figure 11:**
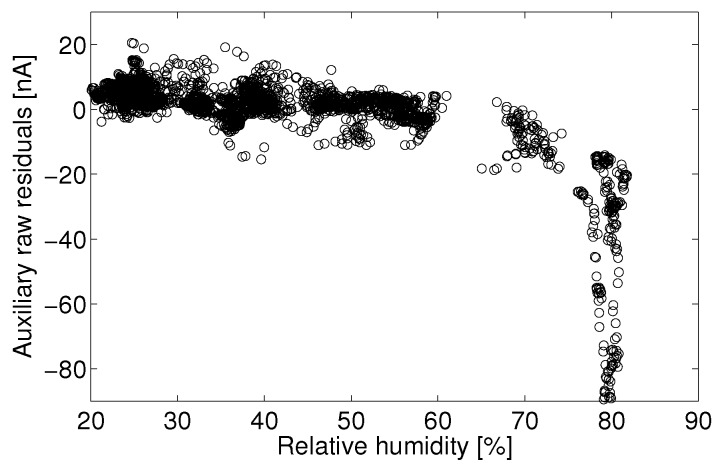
Residual error for a linear least-square fit of the auxiliary electrode current *versus* reference NO measurements between 18 °C and 20 °C for the month of August.

First, consider the auxiliary electrode response to humidity. Figure 11 shows the auxiliary electrode signal against relative humidity for the same data as Figure 10. It can be seen that both the main electrode and the auxiliary electrode show similar response characteristics with respect to humidity. As such, the auxiliary electrode may be an especially useful tool for dealing with humidity levels above 75% Rh.

**Figure 12:**
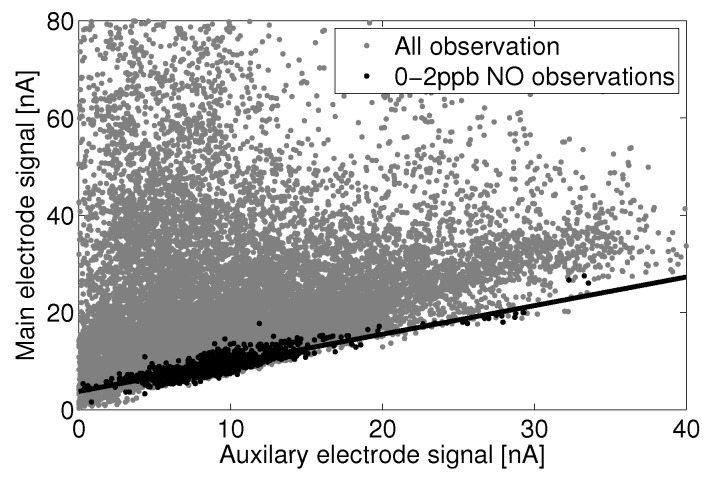
Linear fit of the Alphasense B4-NO main electrode *versus* the auxiliary electrode for a subset of zero current observations.

Figure 12 shows the main electrode signal against the auxiliary electrode signal for all observations and those that are concurrent within reference instrument observations of 0 to 2 ppb. It appears that the auxiliary electrode has slightly higher sensitivity to confounding factors than the main electrode, and the main electrode has a baseline current offset in clean air, which is not observed in the auxiliary electrode.

## 4. Complete Prediction Model

Equations (15) and (16) (for use with the auxiliary signal) give two generalizable models for the Alphasense B4 sensors, where *C* is the predicted concentration, *I* is the measured main electrode current, $I_{z}$ is the measured auxiliary electrode current and $T_{k}$ and $T_{c}$ are the measured temperature in Kelvin and Celsius, respectively. Both models define the main signal current as being the function on a baseline zero current plus the sensitivity of the sensor acting on the concentration of the target analyte: *I* = *Sensitivity*×*Concentration*+*ZeroOffset* . Here, the denominator represents the sensitivity dependence on temperature, and the quantity subtracted from *I* represents the zero current dependence on temperature. Recall the model form for both the zero current (Section 3.2, Equation (9)) and sensitivity (Section 3.1, Equation (8)) as they pertain to Equation (15). The term subtracted from *I* in Equation (16), $\left(b_{1}+b_{2}*I_{z}\right)$, represents a linear scaling and offset of the auxiliary current signal, effectively defining the zero current as being proportional to the auxiliary electrode signal. The denominator in Equation (16) is the same as in Equation (15) and represents the temperature dependence of the main electrode current on sensitivity as per Equation (8). The optimization algorithm used to fit the model form to these data produced better fits when the temperature for the sensitivity term is in units of Celsius, hence the use of both the $T_{k}$ and $T_{c}$ variables.

(15) $$C=\frac{I-a_{1}*exp\left(a_{2}/T_{k}\right)}{a_{3}+a_{4}*exp\left(a_{5}*T_{c}\right)}$$

(16) $$C=\frac{I-(b_{1}+b_{2}*I_{z})}{b_{3}+b_{4}*exp\left(b_{5}*T_{c}\right)}$$

### 4.1. Model 1

**Figure 13:**
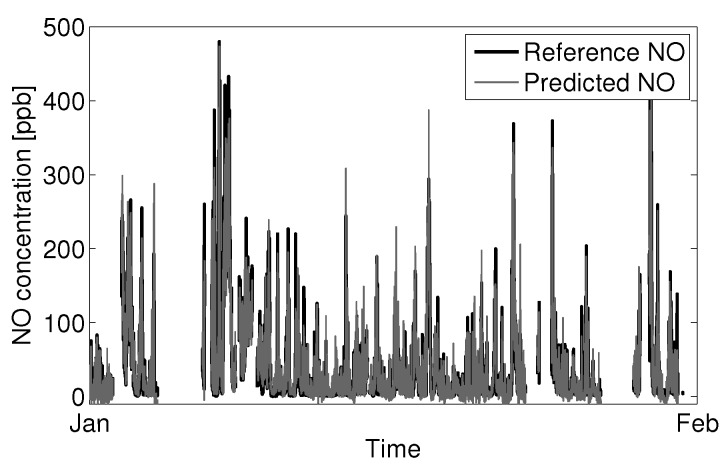
Prediction of NO from the Alphasense B4-NO sensor and reference NO measurements for January.

Applying Equation (15) to January NO data for Sensor 1 with the coefficient values previously determined in fitting the data subsets (Section 3.1 and Section 3.2) gives Figure 13:

The predicted NO concentration shows good agreement with the reference instrument. The RMSE for the above prediction is 14.6 ppb NO. A multivariate fit of Equation (15) to the reference data gives a slightly better optimized fit with an RMSE of 13.6 ppb NO.

Taking the model coefficients from the multivariate fitting of Equation (15) to January data and applying the model backwards to December and forwards to February gives good agreement between prediction and reference with RMSEs of 13.2 ppb and 23.5 ppb NO, respectively. The higher RMSE observed in February seems to be correlated with the large number of fast transient NO events. The electrolytic sensors will have a dampened response to fast transient concentrations because of the sensor’s limitations in response time, resulting in an under-prediction of concentration for these events.

**Figure 14:**
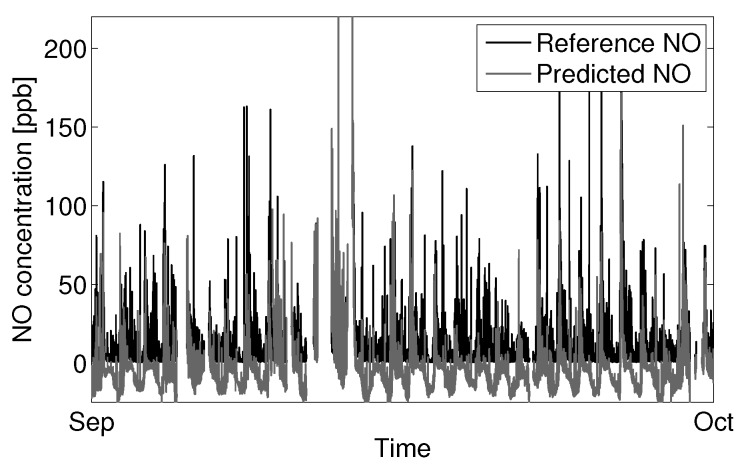
Prediction of NO for September using an optimized model fit against reference NO observations from January.

**Figure 15:**
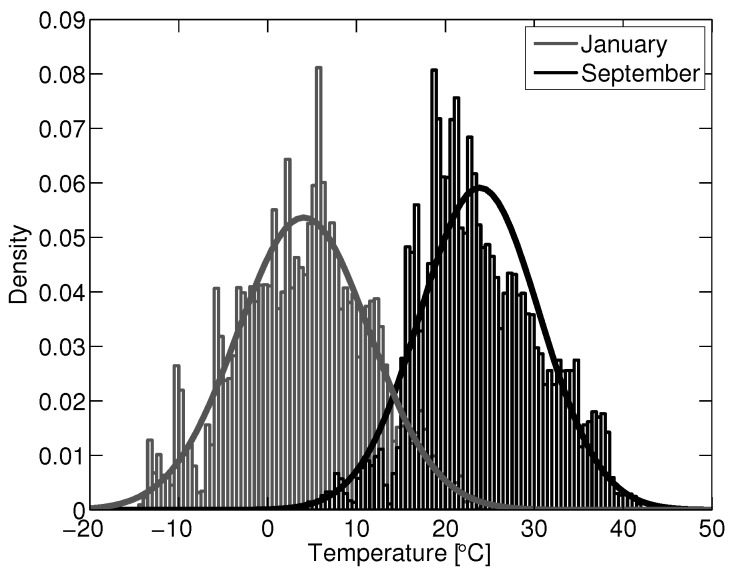
Ambient temperature variable space distributions for January and September.

Figure 14 shows the same model applied to ambient data nine months later (September), with an RMSE of 21.3 ppb NO and a noticeable diurnal trend in error. The predicted concentration appears to capture some spikes in NO, but does poorly to predict the lower background levels of NO, which were well predicted in previous data. Plotting the error for the September data against temperature shows a distinct offset at higher temperatures, which was not observed in the January dataset. This would suggest that the error in the September predictions is primarily due to extrapolating from the January temperature variable space. The variable space is here defined to be the distribution of a variable’s value within a given time series of data. The variable spaces of concern are for those variables that confound the sensors signal. Evaluating a model in an environment outside its variable space will often produce poor results due to extrapolation error. Figure 15 shows the temperature distribution for January and September. It can be seen that the two spaces together span the range of temperatures normally encountered in a moderate ambient environment, thus forming a good variable space for optimized model fitting with respect to temperature. Fitting a model to one space alone will likely always yield poor results when extrapolating to the other.

**Figure 16:**
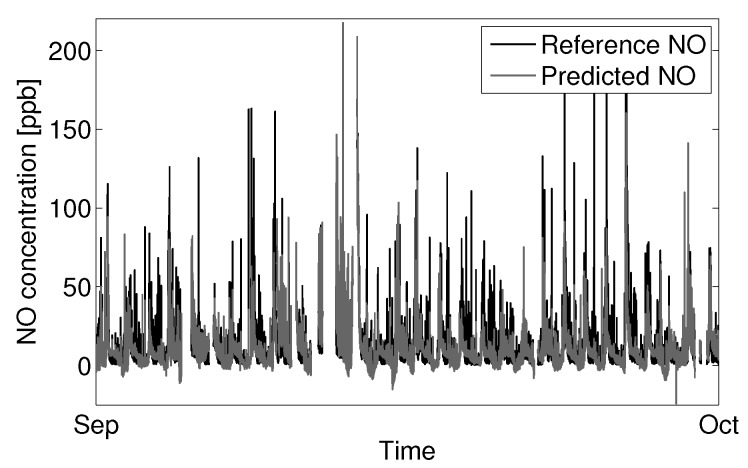
Prediction of NO for September data using an optimized model fit to the aggregate of reference NO observations from January and September.

Fitting the model to the aggregate set of January and September data gives better agreement with predicted values across the larger variable space. Figure 16 shows September data with the model parameters from the aggregate fit, with an RMSE of 12.1 ppb NO. The RMSE of the January prediction increases slightly from to 13.6 ppb NO to 14.9 ppb NO when using a fit to the aggregate dataset.

When inspecting the error from the long-term data (January to September), the error distribution from September appears slightly shifted from that of January, the mean error value differing by −5.3 ppb NO, with error standard deviations for each set being 11.7 ppb and 14.7 ppb NO, respectively. Both the September and January error distributions are normally distributed as tested by the Kolmogorov–Smirnov test at the 95% significance level. The offset in the error could indicate moderate sensor decay and drift over time or may be an artifact of minimizing the fit residual across the two datasets. Regardless, the difference in mean error over a nine-month period is within the standard deviation of a given month’s error. These finding would suggest that the long-term stability of these sensors is suitable for many ambient monitoring applications, as no discernible drift occurred over more than nine months of continuous field use.

Table 2 shows the model coefficients for the model fit for the three sensors.

**Table 2:** Equation coefficients for the optimized fit of Model 1 using the aggregate of reference NO observations from January and September.

$a_{1}$	$a_{2}$	$a_{3}$	$a_{4}$	$a_{5}$
302.02	−1032.17	0.35	0.0055	0.30

### 4.2. Model 2

Using Equation (16) with the auxiliary electrode signal gives similarly promising results with a moderate advantage of better correction at high humidity and superior model fitting with a smaller variable space.

Dynamically fitting the model parameters for Equation (15) to the January data (observations of less than 75% Rh) gives a predicted NO concentration with RMSE of 12.7 ppb NO. Applying the same model parameters to the September data, analogous to Figure 14 for Model 1, gives a prediction with RMSE of 12.1 ppb NO. Unlike Model 1, Model 2 is superior when extrapolating from the temperature variable space used to fit the optimal model parameters. Fitting Equation (16) to the aggregate dataset of January and September data gives RMSEs of 12.7 ppb and 14.1 ppb, respectively.

**Figure 17:**
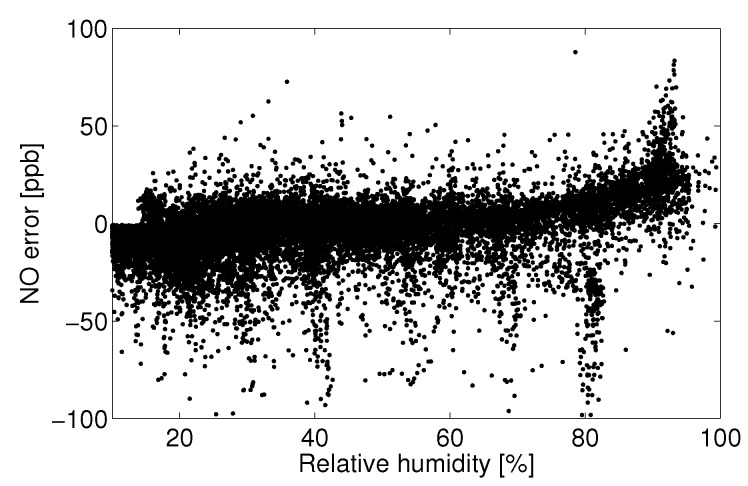
Alphasense B4-NO residual error for the Model 2 fit plotted against humidity.

The error from fitting the aggregate model to all observations from September (all humidity levels) is given in Figure 17. While initial investigation showed that the auxiliary electrode covaries with the main electrode during periods of elevated humidity, these new findings suggest that the auxiliary electrode correction to the main electrode does not provide an advantage over Model 1 during periods of elevated humidity.

**Table 3:** Equation coefficients for the optimized fit of Model 2 using the aggregate of reference NO observations from January and September.

$b_{1}$	$b_{2}$	$b_{3}$	$b_{4}$	$b_{5}$
4.94	0.47	0.36	0.0013	0.35

**Figure 18:**
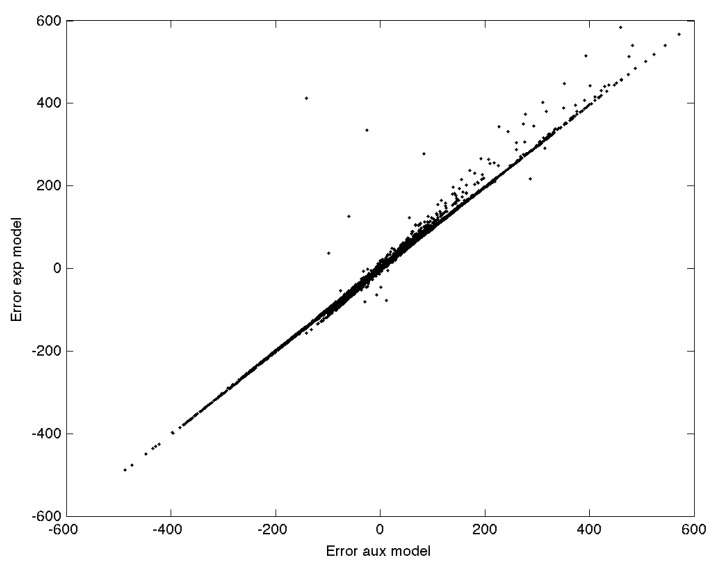
Alphasense B4-NO residual error for predictions from Model 1 *versus* predictions for Model 2 for all observations between December and September.

Applying the models to the whole time series (December to September), using parameters from the optimized fit to the aggregate January/September data, gives an RMSE of 21.9 ppb and 22.3 ppb NO for model Equations (15) and (16), respectively. Table 3 gives the model coefficients for the optimized fit to the joint December and September dataset for Model 2. Figure 18 shows the model error between Model 1 and Model 2 for the same time series of observations between December and September. If the error in the two models were due to independent random processes, then there would be no distinct correlation between the errors in the two models, and averaging the results from the two models would yield better results than any one model alone. A distinct linear trend is evident, however, between the error of the two models. As such, averaging the predicted NO concentration from the two models does little to improve the prediction.

## 5. Conclusions

Electrolytic sensors demonstrate a predictable response to their target analyte when the sensor signals are corrected for confounding environmental factors. This study found little inter-sensor variability among the Alphasense B4 sensors (acceptable variability for most ambient monitoring), implying ease of deployment without unit-specific calibrations. The two models explained here show similar performance when fit to a reference dataset. The first model, which does not use the Alphasense B4-specific auxiliary electrode, showed poor performance when extrapolating from the variable space used to fit the model. The second model, however, which uses the auxiliary electrode as a baseline correction, showed similar performance to the first model (fit to the entire dataset) when extrapolating from the fitting variable space.

In this work, the models were only demonstrated for nitric oxide, but it is reasonable to assume that the model forms are applicable across electrolytic sensors for different types of gas species. A model using the auxiliary electrode is generally the fastest to implement, since the reference data used for deriving model parameters can be shorter in length (and limited in variable space), but still suitable for extrapolating out of that variable space. These findings suggest that a model without auxiliary electrode (Model 1) should only be used if its parameters are derived by fitting the model to the variable space within which the sensor will be used. This may prove an inconvenience if using a collocation method and if one wishes to use one model across many climates. In this case, the sensor will need to be deployed for a sufficient period of time to cover the variable space (*i.e.*, across seasons). The model parameters, however, need only to be derived once, after which it is applicable to all sensors of a given make and model if those sensors exhibit acceptable inter-sensor variability. If reference measurements for other cross-sensitive gas species are present, they may be used as an additional correction factor, treated as contributing to the sensor current in proportion to the measured reference concentration. A temperature dependence on the sensitivity to other cross-sensitive species may also be included for greater fidelity. It is important to note that different sensor types experience different levels of cross-sensitivity to confounding gas species, and site locations will have varying mixes of confounding species. Care should also be taken to minimize changes in airflow over the sensor, as this will introduce changes in the sensor baseline and sensitivity.
